# Variations in Mitochondrial Respiration Differ in IL-1ß/IL-10 Ratio Based Subgroups in Autism Spectrum Disorders

**DOI:** 10.3389/fpsyt.2019.00071

**Published:** 2019-02-20

**Authors:** Harumi Jyonouchi, Lee Geng, Shannon Rose, Sirish C. Bennuri, Richard E. Frye

**Affiliations:** ^1^Department of Pediatrics, Saint Peter's University Hospital, New Brunswick, NJ, United States; ^2^Robert Wood Johnson Medical School-Rutgers, New Brunswick, NJ, United States; ^3^Arkansas Children's Research Institute, Little Rock, AR, United States; ^4^Department of Pediatrics, University of Arkansas of Medical Sciences, Little Rock, AR, United States; ^5^Department of Child Health, University of Arizona College of Medicine-Phoenix, Phoenix, AZ, United States; ^6^Barrow Neurological Institute at Phoenix Children's Hospital, Phoenix, AZ, United States

**Keywords:** autism spectrum disorders, IL-1ß/IL-10 ratio, mitochondrial respiration, monocytes, peripheral blood mononuclear cells (PBMCs)

## Abstract

Autism spectrum disorder (ASD)^7^ is associated with multiple physiological abnormalities, including immune dysregulation, and mitochondrial dysfunction. However, an association between these two commonly reported abnormalities in ASD has not been studied in depth. This study assessed the association between previously identified alterations in cytokine profiles by ASD peripheral blood monocytes (PBMo) and mitochondrial dysfunction. In 112 ASD and 38 non-ASD subjects, cytokine production was assessed by culturing purified PBMo overnight with stimuli of innate immunity. Parameters of mitochondrial respiration including proton-leak respiration (PLR), ATP-linked respiration (ALR), maximal respiratory capacity (MRC), and reserve capacity (RC) were measured in peripheral blood mononuclear cells (PBMCs). The ASD samples were analyzed by subgrouping them into high, normal, and low IL-1ß/IL-10 ratio groups, which was previously shown to be associated with changes in behaviors and PBMo miRNA expression. MRC, RC, and RC/PLR, a marker of electron transport chain (ETC) efficiency, were higher in ASD PBMCs than controls. The expected positive associations between PLR and ALR were found in control non-ASD PBMCs, but not in ASD PBMCs. Higher MRC, RC, RC/PLR in ASD PBMCs were secondary to higher levels of these parameters in the high and normal IL-1ß/IL-10 ratio ASD subgroups than controls. Associations between mitochondrial parameters and monocyte cytokine profiles differed markedly across the IL-1ß/IL-10 ratio based ASD subgroups, rendering such associations less evident when ASD samples as a whole were compared to non-ASD controls. Our results indicate for the first time, an association between PBMC mitochondrial function and PBMo cytokine profiles in ASD subjects. This relationship differs across the IL-1ß/IL-10 ratio based ASD subgroups. Changes in mitochondrial function are likely due to adaptive changes or mitochondrial dysfunction, resulting from chronic oxidative stress. These results may indicate alteration in molecular pathways affecting both the immune system and mitochondrial function in some ASD subjects.

## Introduction

Autism spectrum disorder (ASD) is defined by behavioral symptomatology which results in a heterogeneous phenotype. ASD is also known to be associated with various co-morbid medical conditions, most notably gastrointestinal (GI) symptoms, and sleep disorders (1, 2).

We have previously identified a subset of ASD subjects who exhibit innate immune abnormalities (3). Our recent study revealed that the IL-1ß/IL-10 ratios produced by PBMo may serve as a marker of immune activation or immune mediated inflammation in individuals with ASD. Deviated IL-1ß/IL-10 ratios were seen in up to 40% of ASD subjects in our previous study (4). IL-1ß is a representative inflammatory cytokine, and IL-10 is a representative counter-regulatory cytokine. Thus, IL-1ß/IL-10 ratios are thought to reflect the balance of inflammatory vs. counter-regulatory responses by monocytes.

One knowledge gap in understanding the pathology of ASD is how the various medical comorbidities are related to each other and to abnormalities in brain function. Research has pointed to abnormalities in cellular systems that are common to cells in many organ systems. For example, studies have associated ASD with abnormalities in mitochondrial (5) and redox metabolism (6) that are systems important to varying extents to almost every cell in the body. Other possible connections between different organ systems include abnormalities in cellular regulatory pathways, such as those controlled by microRNA (miRNA). This is a prime area of research as we are learning that abnormalities in miRNA expressions are associated with a wide range of psychiatric diseases, including ASD (7). On the other hand, little is known regarding what changes in miRNA expression are linked to both innate immune and mitochondrial abnormalities in ASD and other neuropsychiatric disorders.

Our previous studies showed that changes in expression of miRNA in ASD PBMo differed in ASD subgroups when they were subdivided on the basis of the IL-1ß/IL-10 ratio produced by PBMo (high, low, and normal as defined in the method section) (4, 8). Namely, many miRNA were up-regulated in the high ratio ASD subgroup, while in the low ratio ASD subgroup, multiple miRNAs were down-regulated, as compared to non-ASD controls (4). Determining targeted genes by these miRNAs indicated that the changes in miRNA expression are expected to affect the key signaling pathways, including RAS, MAPK, and PI3K-AKT pathways: these signaling pathways regulate immune cell differentiation and activation, partly through altering metabolism. Interestingly, this analysis also revealed that changes in miRNA would affect the expression of molecules important for formation of synaptic junctions (4). These results may indicate that there exist alterations in the signaling pathways affecting both the nervous and the immune systems in the high and low IL-1ß/IL-10 ratio ASD subgroups. The above described signaling pathways are also known to affect mitochondrial functions. For example, activation of MAPK and PI3K-AKT pathways enhances glycolysis, but reduces mitochondrial fitness, rendering cells more vulnerable to oxidative stress (9). Therefore, there may be a possibility that mitochondrial function is altered in the high/low IL-1ß/IL-10 ratio ASD subgroups. miRNAs secreted from innate immune cells including monocytes serve as mediators of innate immune responses (10). In fact, platelets and PBMo are major secretary source of miRNAs in the serum. Secreted miRNAs are taken up by other cells and regulate their cellular functions (10). Therefore, changes in IL-1ß/IL-10 ratios by ASD PBMo can be reflected in mitochondrial dysfunction in multiple cell lineages in ASD.

Independent of studies linked to immune abnormalities in ASD, there is also mounting evidence of mitochondrial dysfunction and chronic oxidative stresses in multiple cell types in ASD (11–14). However, primary mitochondrial diseases with known gene mutations are rarely found in ASD subjects (5, 15). Nevertheless, mitochondrial dysfunctions have been reported in 30–80% of individuals with ASD (16), and evidence of oxidative stress may be diagnostic for ASD (17). Putative alterations in signaling pathways as described above, if present, could make some ASD subjects vulnerable to both immune mediated inflammation and mitochondrial dysfunction. However, little is known regarding associations between monocyte cytokine profiles and mitochondrial dysfunction in ASD and other neuropsychiatric diseases.

On the basis of findings described above, we postulated that changes in monocyte cytokine profiles, especially IL-1ß/IL-10 ratios in ASD PBMo, are associated with alterations in mitochondrial respiration. This study examines our hypothesis by evaluating associations between IL-1ß/IL-10 ratios produced by PBMo and variations in mitochondrial respiration in PBMCs, a mixture of multiple lineage cells, in ASD subjects. Associations between mitochondrial parameters and other monocyte cytokines were also determined, since IL-1ß and IL-10 production were often closely associated with production of inflammatory and counter-regulatory cytokine produced by monocytes. Typically developing (TD), age-matched non-ASD subjects served as controls.

## Materials and Methods

### Study Subjects

The study followed the protocol approved by the Institutional Review Board at our institution, Saint Peter's University Hospital, New Brunswick, NJ, United States. In this study, both ASD and non-ASD, TD control subjects were enrolled, and the signed consent forms were obtained prior to entering the study. Consent was obtained from parents if participant was a minor (< 18 years old) or parents had custody. For ASD subjects, we also assessed whether they had history of FA, asthma, allergic rhinitis (AR), and specific antibody deficiency (SAD), or seizure disorders. Any ASD or non-ASD subject diagnosed with chromosomal abnormalities or other genetic diseases, or well-characterized chronic medical conditions involving major organs was excluded from the study. Subjects with common minor medical conditions such as AR, mild to moderate asthma, eczema were not excluded from the study.

#### ASD Subjects

ASD subjects (*N* = 112) were recruited in the Pediatric Allergy/Immunology clinic. The ASD diagnosis was based on the Autism Diagnostic Observation Scale (ADOS) and/or Autism Diagnostic Interview-Revisited (ADI-R), and other standard measures at various autism diagnostic centers, including ours. ASD subjects were also evaluated for their behavioral symptoms and sleep habits with the Aberrant Behavior Checklist (ABC) (18) and the Children's Sleep Habits Questionnaires (CSHQ) (19), respectively. Information regarding cognitive activity and adaptive skills were obtained from previous school records, which documented cognitive ability (by standard measures such as Woodcock-Johnson III test), and adaptive skills (by standard measures such as Vineland Adaptive Behavior Scale (VABS) (20). These were data documented within 1 year of enrollment to the study.

#### Non-ASD Controls

TD, non-ASD control subjects (*N* = 38) were recruited from the pediatric Allergy/Immunology and General Pediatrics Clinics. These subjects were not reported to have any medical conditions included in the exclusion criteria and self-reported not to have seizure disorders or known immunodeficiency.

Demographic information of the study subjects is summarized in Table 1. There were no differences between females and males by two tailed Mann-Whitney test with regard to mitochondrial respiration parameters and monocyte cytokine profiles examined in this study.

**Table 1:** Demographics of ASD children.

	**ASD^**a**^ subjects (*N* = 112)**	**Non-ASD controls (*N* = 38)**
**AGE (YEARS)**		
Median (range)	12.3 (2.5–30.3)	13.4 (3.9–29.7)
Mean ± SD	12.6 ± 5.9	13.8 ± 7.2
Gender (M:F and %)	97:15 (86.6%: 13.4%)	26:12 (68.4%: 31.4%)
Ethnicity	AA 6, Asian 21, Mixed 2, C 83	Asian 3, Mixed 3, C 32
Cognitive activity < 1%	83/112 (74.1%)	0
Disturbed sleep	34/112 (30.4%)	N/A
GI symptoms	75/112 (67.0%)	Unknown^c^
Seizure disorders	14/112 (12.5%)	0
Specific antibody deficiency	18/112 (16.1%)	0
Asthma^b^	12/112 (10.7%)	Unknown
Allergic rhinitis^b^	23/112 (20.5%)	Unknown

a *AA, African American; ASD, autism spectrum disorder; C, Caucasian; N/A, not applicable; SD, standard deviation*.

b *Frequencies of asthma and allergic rhinitis are equivalent for those reported in general population*.

c No self-reported GI complaint by non-ASD controls

#### Diagnosis of FA

IgE mediated FA was diagnosed with reactions to offending food, by affecting skin, GI, and/or respiratory tract immediately (within 2 h) after intake of offending food, supported by prick skin testing (PST) reactivity, and/or presence of food allergen-specific IgE in the serum. NFA was diagnosed if GI symptoms resolved, following implementation of a restricted diet (i.e., avoidance of offending food), and symptoms recurred upon re-introduction of offending food, following the Food Allergy Diagnostic Guidelines (21). NFA patients are per definition, non-reactive to PST, and negative for food allergen-specific, serum IgE (21).

#### Diagnosis of Asthma and Allergic Rhinitis

AR and allergic conjunctivitis (AC) were diagnosed with positive PST reactivity, and/or presence of allergen-specific IgE in the serum, accompanied by clinical features consistent with AR and AC (22, 23). Asthma diagnosis was based on the guidelines from the Expert Panel Report 3 (24). Asthma, without PST reactivity to allergens and/or allergen-specific IgE antibodies was categorized as non-atopic asthma (23).

#### Specific Antibody Deficiency (SAD)

SAD was diagnosed by the absence of protective levels of antibody (Ab) titers (>1.3 μg/mL) to more than 11 of 14 serotypes of *Streptococcus pneumonia*, following a booster dose of Pneumovax® (25) or PCV13, a standard diagnostic measure for SAD.

### Sample Collection

Blood samples were obtained by venipuncture after obtainment of informed consent. Efforts were made to obtain the samples at the time of medically required blood work to minimize the numbers of venipuncture. For the non-ASD control subjects, only 1 sample was obtained. For select ASD subjects, samples were obtained at 2–3 time points to assess variability of parameters that we tested. Venipuncture was conducted by the physician and if requested, the site of venipuncture was numbed by applying a topical lidocaine/prilocaine cream (Emla cream®).

### Cell Cultures

PBMCs were isolated by Ficoll-Hypaque density gradient centrifugation. PBMo were purified by negatively selecting PBMo depleting T, B, natural killer, and dendritic cells from PBMCs, using magnetic beads labeled with anti-CD3, CD7, CD16, CD19, CD56, CD123, and glycophorin A (monocyte separation kit II—human, MILTENYI BIOTEC, Cambridge, MA, United States).

PBMo cytokine production was assessed by incubating purified PBMo (2.5 × 10^5^ cells/ml) overnight with a TLR4 agonist (LPS; 0.1 μg/ml, GIBCO-BRL, Gaithersburg, MD, USA), a TLR2/6 agonist (zymosan; 50 μg/ml, Sigma-Aldrich, St. Luis, Mo), and a TLR7/8 agonist (CL097, water-soluble derivative of imidazoquinoline, 20 μM, InvivoGen, San Diego, CA, Unites States) in RPMI 1640 with additives as previously described (26). Overnight incubation (16–20 h) was adequate to induce the optimal responses in this setting. The culture supernatant was used for cytokine assays. LPS is a representative endotoxin, reflecting a common pathway of innate immune responses by gram negative [G (–)] bacteria. Zymosan is a representative innate immune stimulus from G (+) bacteria and fungi. CL097 mimics stimuli from ssRNA viruses, common respiratory pathogens causing respiratory infection, such as influenza. These stimuli have been widely used for testing innate immune responses.

Levels of pro-inflammatory [tumor necrosis factor-α (TNF-α), IL-1β, IL-6, IL-12p40, and IL-23] and counter-regulatory [IL-10, transforming growth factor-ß (TGF-ß) and soluble TNF receptor II (sTNFRII)] cytokines were measured by enzyme-linked immuno-sorbent assay (ELISA);10–100 μl/well supernatant were used for ELISA, depending on culture conditions. The ELISA, OptEIA™ Reagent Sets for IFN-γ, IL-1ß, IL-5, IL-6, IL-10, IL-12p40, and TNF-α (BD Biosciences, San Jose, CA, USA), and for sTNFRII, IL-17 (IL-17A), and TGF-ß were obtained from BD Biosciences and R & D (Minneapolis, MN, United States), respectively. IL-23 ELISA kit was purchased from eBiosciences, San Diego, CA, United States. Intra- and inter-variations of cytokine levels were < 5%.

### Categorizing ASD Samples Based on IL-1ß/IL-10 Ratios

Previously, we observed both high and low IL-1ß/IL-10 ratios in subsets of ASD PBMo as compared to non-ASD controls (8). We found changes in IL-1ß/IL-10 ratios were associated with behavioral changes as well as changes in miRNA expression (4, 8). In this study, we also observed the presence of high and low IL-1ß/IL-10 ratios in some ASD PBMo, as compared to control PBMo (Supplemental Figure 1). To assess whether there was an association between IL-1ß/IL-10 ratios produced by PBMo and mitochondrial respiration exhibited by PBMCs, we subdivided ASD samples into subgroups based on the IL-1ß/IL-10 ratios produced by ASD PBMo as described below, following the criteria used in our previous study (4, 8).

#### High IL-1ß/IL-10 Ratio

IL-1ß/IL-10 ratios > +2 standard deviation (SD) than control cells under at least 1 culture condition and/or > +1 SD under more than 2 culture conditions.

#### Normal IL-1β/IL-10 Ratio

IL-1ß/IL-10 ratios between −1 SD < IL-1ß/IL-10 ratios < +1 SD under all the culture conditions, or +1 SD < IL-1ß/IL-10 ratios < +2 SD under only one culture condition.

#### Low IL-1β/IL-10 Ratios

IL-1ß/IL-10 ratios < −1 SD under at least 1 culture condition.

As for ASD subjects whose samples were taken at 2–3 time points, most subjects were categorized in the same group with analysis of samples taken at 2–3 time points. One ASD subject who was assessed at 3 time points revealed high ratios at 2 time points and normal ratio at 1 time point when his GI symptoms became under control. This patient was categorized as the high ratio group. Another subject revealed low ratios at 2 time points and a normal ratio at one time point, thus this subject was categorized as the low ratio group. Most non-ASD controls fall into the normal IL-1β/IL-10 group, except for 2 subjects who fall into the high ratio group and 1 subject who falls into the low ratio group, consistent with our previous study (4). Since our current hypothesis was developed based on this subgrouping definition, we used the same definition in this study.

### Assays of Mitochondrial Function

PBMCs (2 × 10^6^ cells) were suspended in bio-freezing medium (90% heat-inactivated fetal calf serum and 10% DMSO) and kept in −20°C for about 1 h and then transferred to −80°C degree freezer and kept until shipment. Then samples were sent to Dr. R. Frye's laboratory on dry ice where Seahorse Extracellular Flux (XF) 96 Analyzer (Seahorse Bioscience, Inc., North Billerica, MA, United States) was used for measurement of oxygen consumption ratio (OCR), which is an indicator of mitochondrial respiration, in real time in live PBMCs (14, 27). Several measures of mitochondrial respiration, including basal respiration, ALR, PLR, MRC, and RC, were derived by the sequential addition of pharmacological agents to the respiring cells. For each parameter, three repeated rates of oxygen consumption are made over an 18 min period. First, baseline cellular oxygen consumption is measured, from which basal respiration is derived by subtracting non-mitochondrial respiration. Next oligomycin, an inhibitor of complex V, is added, and the resulting OCR is used to derive ALR (by subtracting the oligomycin rate from baseline cellular OCR) and PLR subtracting non-mitochondrial respiration from the oligomycin rate). Next carbonyl cyanide-p-trifluoromethoxyphenyl-hydrazon (FCCP), a protonophore, is added to collapse the inner membrane gradient, driving the ETC to function to its maximal rate, and MRC is derived by subtracting non-mitochondrial respiration from the FCCP OCR. Lastly, antimycin A, a complex III inhibitor, and rotenone, a complex I inhibitor, are added to shut down ETC function, revealing the non-mitochondrial respiration. RC is calculated by subtracting basal respiration from maximal respiratory capacity.

Both ALR and MRC are measures of the ability of the electron transport chain (ETC) to produce ATP, the molecule that carries energy to other areas of the cell to support vital functions. However, the ETC is also a major source of the production of reactive oxygen species (ROS), which can damage the mitochondrial and the cell if produced in excess. The ETC can “leak” some of its energy to reduce ROS production. This “leak” is measured by PLR and makes the ETC less efficient at producing energy. In general, PLR should increase as more ATP is produced since the production of ATP does create ROS. The ratio of the measures of ATP production, specifically ALR and MRC, to PLR can provide a measure of efficiency of the ETC. Theoretically this ratio would be very high with very efficient mitochondrial function and very low in dysfunctional mitochondria where a great amount of ROS is produced to make energy.

### Statistical Analysis

For comparison of two sets of numerical data, two tailed Mann-Whitney test was used. For comparison of several sets of numerical data, a one-way analysis of variance (ANOVA) was used if the data were distributed normally. If the data were not normally distributed, Kruskal-Wallis test was used. For differences in frequency between two groups, Fisher exact test was used. For differences in frequency among multiple groups, Chi-square test and Likelihood ratio were used. Co-variance of repeated measures and a linear association between two variables were assessed by regression analysis (mixed models—repeated measures) and Spearman test, respectively. NCSS12 (Kaysville, UT, United States) was used for analysis. A *p*-value of < 0.05 was considered nominally significant. Statistical measures used in this study are summarized in Supplemental Figure 2.

## Results

### Mitochondrial Respiration in ASD PBMCs

**Figure 1:**
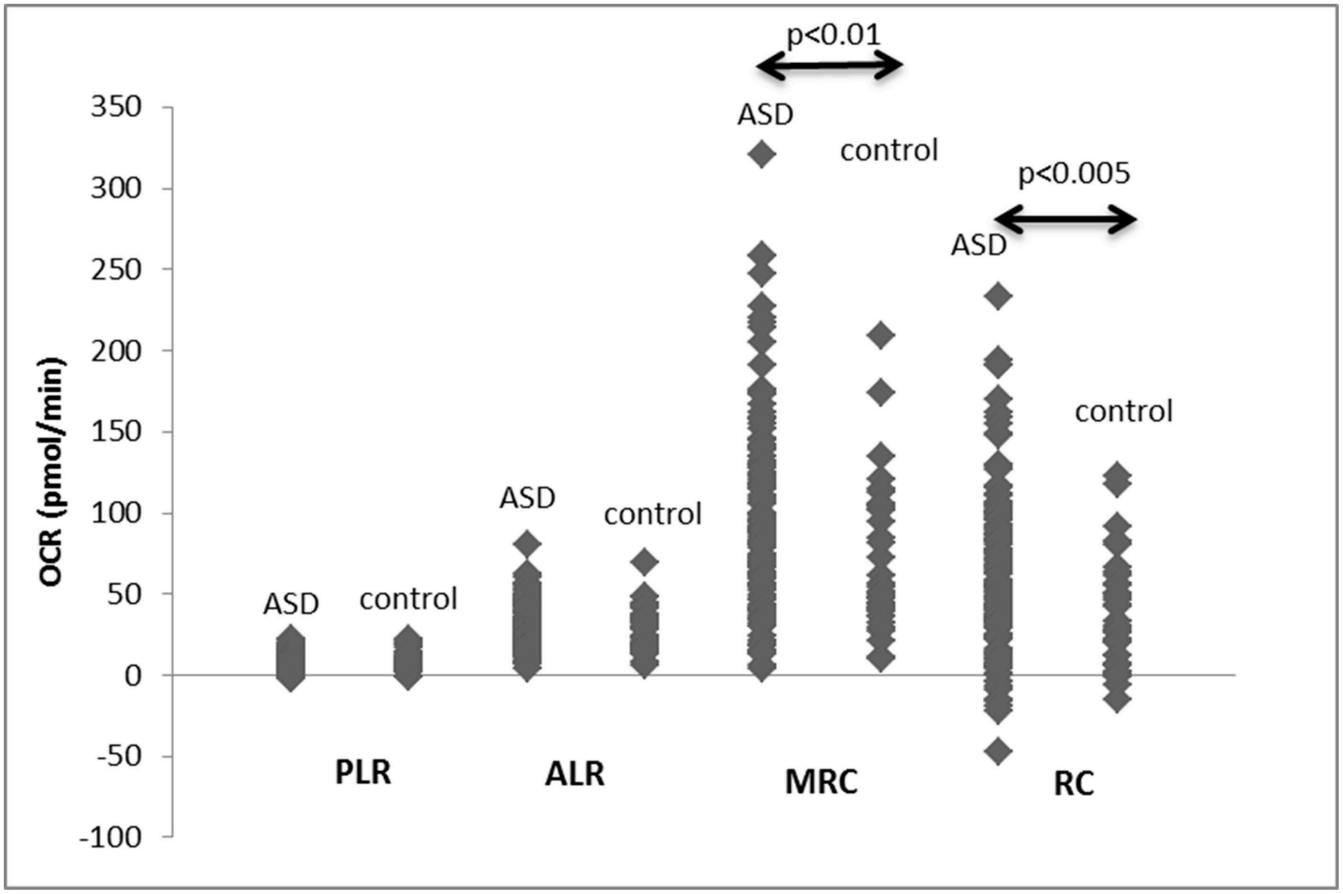
PLR, ALR, MRC, and RC values in PBMCs from ASD subjects as well as control non-ASD subjects. F-ratios are 0.2971 (PLR), 0.2484 (ALR), 8.6833 (*p* < 0.005), and 13.487 (*p* < 0.001) by Welch's test. *P*-values shown in the figure by two-tailed Mann-Whitney test.

When mitochondrial respiration parameters were compared between all the ASD and non-ASD control samples, MRC and RC were higher in ASD cells than non-ASD controls (Figure 1). There were no differences in PLR and ALR between ASD and non-ASD control PBMCs (Figure 1).

**Figure 2:**
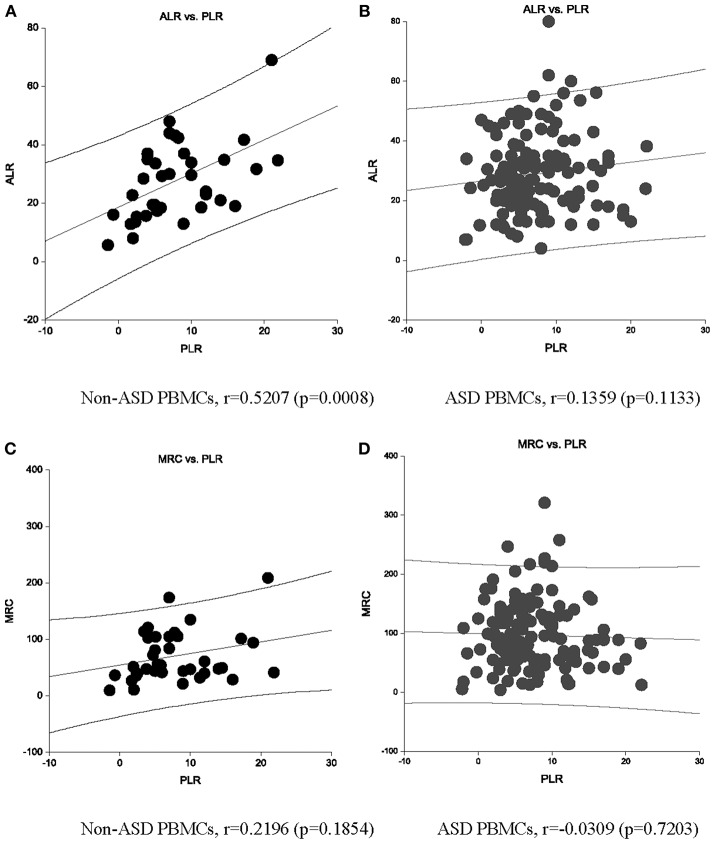
An association between ALR and PLR OCR (pmol/min) are shown in non-ASD **(A)** and ASD **(B)** PBMCs. An association between MRC and PLR OCR (pmol/min) are also shown in non-ASD **(C)** and ASD **(D)** PBMCs. Correlation Co-efficient and *p*-values shown were by Spearman test.

Non-ASD control PBMCs demonstrated the expected positive correlation between PLR and ALR (Figure 2A). MRC and PLR tended to show similar positive correlation, but not statistically significant (Figure 2C). However, these expected positive associations were not observed in ASD PBMCs (Figures 2B,D). For ALR, this was secondary to ASD PBMCs demonstrating low PLR despite high ALR, and those with high PLR demonstrating low ALR (Figure 2B). These cells are likely to have a tightly coupled ETC with very active mitochondrial respiration, or under mitochondrial dysfunction, respectively. Given these findings, we assessed differences in ALR/PLR, MRC/PLR, and RC/PLR ratios, as markers of ETC efficiency, between ASD and non-ASD PBMCs. As shown in Table 2, we observed nominally significant differences in RC/PLR ratios between ASD and non-ASD samples. This is secondary to presence of ASD subjects with high RC/PLR ratios, but some ASD subjects also showed low RC/PLR ratios.

**Table 2:** Mitochondrial respiration ratios in the ASD cell subgroups based on IL-1ß/IL-10 ratios by PBMo.

	**Total ASD samples (*N* = 136)**	**Non-ASD controls (*N* = 38)**	**Statistics (mann-whitney test)**
			
ALR/PLR ratio^a^, ^b^	5.4 ± 11.1	4.9 ± 3.1	*p* = 0.8949
MRC/PLR ratio	19.6 ± 37.7	8.8 ± 14.0	*p* = 0.0558
RC/PLR ratio^b^	13.2 ± 27.1	4.4 ± 8.6	*p* = 0.01239

a *F-ratios are 1.314 (ALR/PLR), 6.693 (MRC/PLR), and 9.009 (RC/PLR) by Welch's test*.

b *ALR, ATP-linked respiration; MRC, Maximum respiration capacity; PLR, proton-leak respiration; RC, reserve capacity*.

### Associations Between Mitochondrial Respiration and Monocyte Cytokine Profiles in ASD

As summarized in Table 3, we found several positive or negative associations in the ASD samples mainly between IL-1ß and IL-6 levels under LPS stimulated cultures, and mitochondrial respiratory parameters. Specifically, negative associations were observed between PLR and IL-1ß/IL-10 ratio and IL-1ß levels under LPS stimulated cultures in ASD samples. On the other hand, no significant associations between these parameters were observed in non-ASD controls, except for associations between MRC and TNF-α (under the zymosan-stimulated cultures) or CCL2 (without stimuli) (Table 3).

**Table 3:** Mitochondrial respiration ratios in the ASD cell subgroups based on IL-1ß/IL-10 ratios by PBMo.

	**Correlation coefficient ASD samples (*N* = 136)**	**Correlation coefficient non-ASD controls (*N* = 38)**
**PLR^c^**		
Ratio (medium)	0.1855 (*p <* 0.05)^a^, ^b^	*0.1133*
Ratio (LPS)	−0.3266 (*p <* 0.0001)	*0.1882*
IL-1ß (LPS)	−0.2742 (*p <* 0.005)	*0.1242*
TGF-ß (zymosan)	0.2067 (*p <* 0.02)	*−0.1636*
**ALR**		
IL-10 (LPS)	0.181 (*p <* 0.05)	0.188
**MRC^d^**		
IL-1ß (LPS)	0.2431 (*p <* 0.005)	0.0119
IL-10 (LPS)	0.251 (*p <* 0.005)	0.0219
IL-6 (medium)	0.1916 (*p <* 0.05)	−0.1224
IL-6 (LPS)	0.2999 (*p <* 0.0005)	0.1286
TNF-α (zymosan)	−0.2278 (*p <* 0.01)	−0.3681 (*p <* 0.05)
CCL2 (medium)	−0.1284	−0.4162 (*p <* 0.01)
**ALR/PLR**		
IL-1ß (LPS)	0.1938 (*p <* 0.05)	−0.1191
IL-6 (LPS)	0.1954 (*p <* 0.05)	0.3046
**MRC/PL**		
Ratio (LPS)	0.2034 (*p <* 0.02)	−0.1061
IL-1ß (LPS)	0.2462 (*p <* 0.005)	−0.1417
IL-6 (LPS)	0.2263 (*p <* 0.01)	0.1668

a *Values of Correlation coefficients revealed significant results in ASD and control samples are shown*.

b *Correlation coefficient by Spearman test; Statistically significant values are shown with p-values*.

c *ALR, ATP-linked respiration; LPS, lipopolysaccharide; MRC, Maximum respiration capacity; PLR, proton-leak respiration; RC, reserve capacity*.

d *Correlations between RC or RC/PLR and monocyte cytokine levels are almost identical as observed in MRC and MRC/PLR vs. monocyte cytokine levels and not included in this table*.

**Figure 3:**
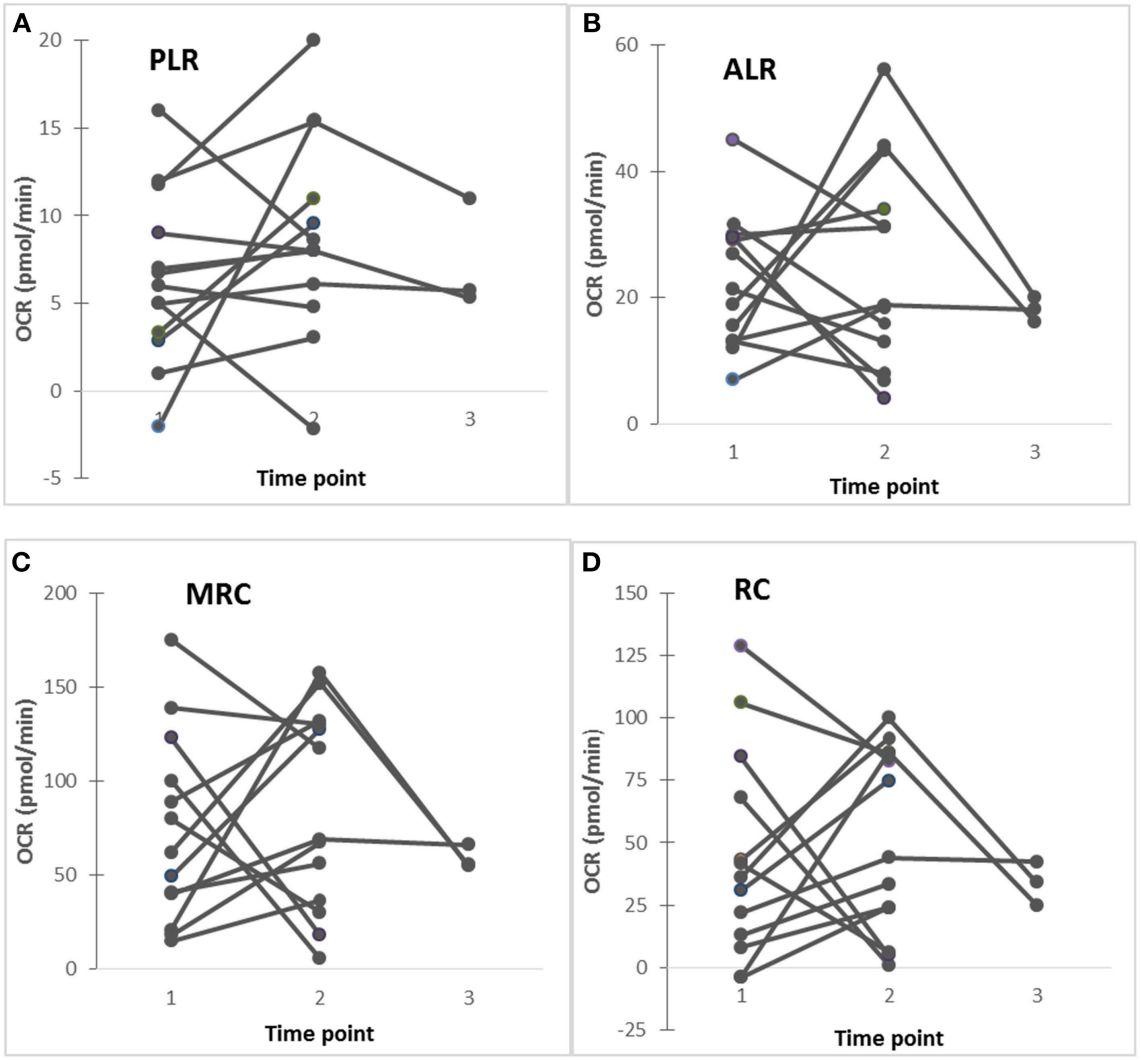
**(A–D)** Changes in mitochondrial respiration (PLR, ALR, MRC, and RC) in ASD subjects studied at 2–3 time points, showing that in some ASD subjects revealed stable these parameters, while others show fluctuating these parameters. Five ASD subjects (4 males and 1 female) showed stable clinical conditions without fluctuating behavioral symptoms, while 8 ASD subjects (7 males and 1 female) revealed fluctuating behavioral symptoms (anxiety, irritability, OCD, and self-injurious behaviors) along with fluctuating GI (diarrhea alternating with constipation) symptoms.

In 13 ASD subjects, mitochondrial respiration was measured at 2–3 time-points along with cytokine profiles by PBMo. Repeated measures regression demonstrated that IL-1ß/IL-10 ratios under CL097 stimulated cultures were positively associated with ALR (*p* = 0.026), MRC (*p* = 0.014), and RC (*p* = 0.0294). In these samples, mitochondrial respiration appeared to change in some ASD subjects, while these values remained stable in others (Figure 3). However, these numbers are too small to confirm this trend and further studies are required.

We also assessed the associations between monocyte cytokine profiles and ALR/PLR, MRC/PLR, and RC/PLR ratios, as markers of ETC efficiency. We observed positive associations mainly between these ETC efficiency markers and IL-1ß and IL-6 levels under LPS stimulated culture conditions (Table 3). The results of association analysis between RC/PLR and monocyte cytokine profiles are almost identical to those between MRC/PLR and cytokine profiles (data now shown). We did not observe significant associations between monocyte cytokine levels and ETC efficiency parameters in non-ASD controls.

### Clinical Features of IL-1ß/IL-10 Ratio Based ASD Subgroups

Clinical features of ASD subjects in the ASD subgroups are summarized in Table 4. We found frequency of history of NFA differed across the ASD subgroups; frequency of history of NFA was higher in the low ratio ASD subgroup than normal ratio group (*p* < 0.05 by Fisher's exact test). Disturbed sleep was reported at a higher frequency in the lower ratio ASD subgroup than in normal ratio ASD subgroup (*p* < 0.05 by Fisher's exact test). No difference was found in frequency in seizures, SAD, asthma, or AR among the IL-1ß/IL-10 ratio based ASD subgroups.

**Table 4:** Demographics and clinical characteristics of the ASD study subjects, when ASD subjects subdivided into the high, normal, and low IL-1ß/IL-10 ratios groups.

	**ASD subjects subgrouped based on IL-1ß/IL-10 ratios**		
	**High ratio (*N* = 51)**	**Normal ratio (*N* = 47)**	**Low ratio (*N* = 14)**
**AGE**			
Median (range)	11.8 year (2.5–30.0)	13.4 year (3.8–30.0)	11.1 year (5.8–19.8)
Mean ± SD	12.3 ± 6.3 year	13.1 ± 5.9 year	11.8 ± 4.9 year
Gender (M:F and %)	43:8 (84.3%: 15.7%)	41:6 (87.2%: 12.8%)	13:1 (92.9%: 7.1%)
Ethnicity	AA^a^ 5, Asian 10, C 36	AA 2, Asian 9, Mixed 2, C 34	AA 1, Asian 2, C 11
Cognitive activity (< 1st %)	37/51 (72.5%)	32/47 (70.2%)	11/14 (78.6%)
Disturbed sleep	16/51 (31.4%)	11/47 (23.4%)	8/14 (57.1%)^c^
GI symptoms^d^	33/51 (64.7%)	32/47 (68.1%)	11/14 (78.6%)
History of NFA	29/51 (56.9%)	21/47 (44.7%)	11/14 (78.6%)^b^
Seizure disorders	5/51 (9.8%)	6/47 (12.8%)	3/14 (21.4%)
SAD	7/51 (14.9%)	9/47 (19.1%)	3/14 (21.4%)
Allergic rhinitis	11/51 (21.6%)	10/47 (21.3%)	2/14 (14.3%)
Asthma	8/51 (11.9%)	4/47 (8.5%)	0

a *AA, African American; ASD, autism spectrum disorder; C, Caucasian; GI, gastrointestinal; NFA, non-IgE mediated food allergy; SAD, specific antibody deficiency; SD, standard deviation*.

b Significantly different in frequencies by Chi-Square test and Likelihood Ratio (p < 0.05). Frequency of history of NFA is higher in the low ratio ASD subgroup than in the normal ratio ASD subgroup (p < 0.05 by Fisher's exact test)

c *Frequency of disturbed sleep was higher in the low ratio ASD subgroup than in the normal ratio ASD subgroup (p < 0.05 by Fisher's exact test)*.

d *GI symptoms present at the time of sample obtainment: Constipation is the most common complaint*.

### Mitochondrial Respiration in IL-1ß/IL-10 Ratio ASD Subgroups

In this study, we found a distribution of ASD PBMo samples into the high, normal, or low IL-1ß/IL-10 ratio subgroups similar to our previous reports (Table 5) (4). Most non-ASD control samples (35/38 sample, 92%) were categorized as normal ratio subgroup. Consistent with our previous results (4), we observed differences in production of monocyte cytokines (IL-6, TNF-α, and CCL2) across the IL-1ß/IL-10 ratio based ASD subgroups (Supplemental Table 1).

**Table 5:** IL-1ß/IL-10 ratios and Mitochondrial function in ASD cells and non-ASD control cells.

	**IL-1ß/IL-10 ratio^a^ based ASD cell subgroups**			**Non-ASD controls (*N* = 38)**	**Krushkal wallis test**
	**High (*N* = 56)^e^**	**Normal (*N* = 59)**	**Low (*N* = 22)**		
**IL-1ß/IL-10 RATIOS CULTURED WITH** ***P*****-VALUE**					
medium	1.54 ± 2.03^d^	0.79 ± 1.12	0.32 ± 0.30	0.88 ± 0.92	< 0.00001
LPS^c^	2.03 ± 1.43	1.19 ± 0.51	0.77 ± 0.56	1.81 ± 2.02	< 0.00001
Zymosan	5.86 ± 3.94	2.53 ± 0.87	1.42 ± 0.74	3.25 ± 1.98	< 0.00001
CL097	9.40 ± 16.84	2.89 ± 2.84	2.72 ± 2.19	3.95 ± 2.77	0.00061
**MITOCHONDRIAL RESPIRATION^b^** ***P*****-VALUE**					
PLR	6.2 ± 5.2	8.3 ± 5.2	7.5 ± 3.8	7.9 ± 5.8	0.06771
ALR	27.2 ± 10.8	31.2 ± 14.0	27.3 ± 15.7	27.7 ± 13.2	0.5153
MRC^f^	93.3 ± 57.2	104.7 ± 59.0	84.6 ± 59.3	70.8 ± 44.4	0.0269
RC	59.9 ± 49.5	64.6 ± 48.5	49.7 ± 47.9	35.1 ± 33.6	0.00788

a *IL-1ß/IL-10 ratios are those obtained from purified ASD monocytes cultured with medium only, LPS (0.1 μg/ml), zymosan (50 μg/ml), or C097 (20 μM) as detailed in the materials and methods section. IL-1ß/IL-10 ratios were calculated as IL-1ß levels/IL-10 levels in each culture condition. The high ratio group revealed higher ratios than normal and low ratio groups under all the culture conditions than the normal and low ratio groups (p < 0.005 by two tailed Mann-Whitney test). The normal ratio group also revealed higher ratios than the low ratio group under the culture condition tested (p < 0.005, by two tailed Mann-Whitney test), except for the cultures under CL097*.

b *Mitochondrial respiration parameters were measured in PBMCs*.

c *Abbreviations used; ALR, ATP-linked respiration; LPS, lipopolysaccharide; MRC, Maximum respiration capacity; PLR, proton-leak respiration; RC, reserve capacity*.

d *All the data are expressed as a mean value ± SD*.

e *In 10 ASD subjects, samples were obtained at 2 time points, and in 3 ASD subjects. samples were obtained at 3 time points*.

f *MRC and RC were higher than non-ASD controls in the high (p < 0.05 for MRC, p < 0.02 for RC), and normal (p < 0.005 for both MRC and RC) ratio ASD subgroups by two tailed Mann Whitney test*.

Given these results, we then assessed mitochondrial parameters in the IL-1ß/IL-10 based ASD subgroups as summarized in Table 5. MRC and RC differed among the ASD subgroups. Specifically, the high and normal IL-1ß/IL-10 ratio ASD subgroups revealed a higher MRC and RC than non-ASD controls. MRC and RC in the low ratio ASD subgroup did not differ from those in non-ASD controls. Parameters of ETC efficiency (ALR/PLR, MRC/PLR, and RC/PLR ratios) were also assessed in these ASD subgroups. Only RC/PLR ratios revealed changes in association with the IL-1ß/IL-10 ratio based ASD subgrouping (Table 6). Specifically, the high and normal ratio ASD subgroups revealed higher RC/PLR ratios than non-ASD controls.

**Table 6:** Mitochondrial respiration ratios in the ASD cell subgroups based on IL-1ß/IL-10 ratios by PBMo.

	**IL-1ß/IL-10 ratio**			**Non-ASD controls (*N* = 38)**	**Statistics (krushkal-wallis test)**
	**High (*N* = 56)**	**Normal (*N* = 59)**	**Low (*N* = 22)**		
**ALR/PLR RATIO^a^**					
	4.3 ± 11.1^c^	6.8 ± 12.6	4.7 ± 4.8	*4.9 ± 3.1*	*p = 0.9186*
**MRC/PLR RATIO**					
	17.3 ± 38.5	23.3 ± 42.0	15.6 ± 20.0	8.8 ± 14.0	*p =* 0.1583
**RC/PLR RATIO^b^**					
	12.0 ± 27.7	15.6 ± 30.0	9.8 ± 15.3	4.4 ± 8.6	*p =* 0.0430

a *F-ratios are 0.7654 (p = 0.9153), 1.4245 (p = 0.1040), 3.559 (p = 0.0229) for ALR/PLR, MRC/PLR, and RC/PLR ratios, respectively, by Welch's test among the IL-1ß/IL-10 ratio ASD subgroups*.

b *RC/PLR ratios differ among the ASD subgroups and non-ASD controls. Specifically, the RC/PLR ratios are higher in the ASD high (p < 0.05) and normal (p < 0.01) ratio groups than non-ASD controls (by two-tailed Mann-Whitney test)*.

c *The results were expressed as a mean ±SD*.

### Associations Between Mitochondrial Respiration Parameters and Monocyte Cytokine Profiles in the IL-1ß/IL-10 Based ASD Subgroups

As shown in Table 4, ASD samples as a whole, revealed associations only between PLR and IL-1ß/IL-10 ratios. In contrast, in the IL-1ß/IL-10 based ASD subgroups, ALR and MRC revealed significant associations with the IL-1ß/IL-10 ratios, but not PLR (Table 7). Moreover, associations differed across the IL-1ß/IL-10 based ASD subgroups. Since MRC and RC revealed almost identical results, in this analysis, the results of associations between RC and IL-1ß/IL-10 ratios are not shown in the Table 7.

**Table 7:** Associations between parameters of mitochondrial respiration (PLR, ALR, and MRC) and IL-1ß/IL-10 ratios by PBMo under various culture conditions.

**IL-1ß/IL-10 ratio correlation with PLR, ALR, and MRC^a^**	**IL-1ß/IL-10 ratio**^d^ **based ASD subgroups**			**Non-ASD control cells (*N* = 38)**
	**High ratio (*N* = 56)**	**Normal ratio (*N* = 59)**	**Low ratio (*N* = 22)**	
**PLR^c^**	**No association**	**No association**	**No association**	**No association**
**ALR**				
Ratio (medium)	−0.4379 (*p <* 0.001)^b^	−0.0752	0.0594	0.1133
Ratio (LPS)	−0.3638 (*p <* 0.01)	0.3294 (*p <* 0.02)	0.117	0.1882
**MRC^e^**				
Ratio (medium)	−0.3275 (*p <* 0.02)	0.0676	0.0407	0.0783
Ratio (LPS)	−0.1612	0.4935 (*p <* 0.0001)	0.2333 (*p <* 0.005)	−0.1055
Ratio (CL097)	−0.0323	−0.1066	−0.5885 (*p <* 0.005)	−0.264

a *Values of Correlation coefficients revealed significant results in at least one of ASD subgroups or non-ASD controls are shown. Stimulants used for cultures of PBMo are shown in the parentheses*.

b *Correlation coefficient by Spearman test. Statistically significant values are shown with p-values*.

c Abbreviations used: please see Table 3

d *Definition of high, normal, and low ratio groups are detailed in the method section*.

e *Correlations between RC and IL-1ß/IL-10 ratios are almost identical as observed in associations between MRC and ratios. Thus, not shown in the table*.

Multiple cytokines (IL-1ß, IL-10, IL-6, TNF-α, and TGF-ß) under various culture conditions revealed positive or negative associations with mitochondrial parameters in the IL-1ß/IL-10 ratio based ASD subgroups (Table 8). Moreover, associations differed markedly across the IL-1ß/IL-10 ratio based ASD subgroups (Table 8). Again, MRC and RC revealed almost identical associations (data not shown). Specifically, nominally significant associations between PLR and cytokine levels were observed only in the high ratio ASD group (Table 8) and *p*-values are not under 0.005. In contrast, ALR and MRC revealed significant associations with several cytokines across the ASD subgroups (Table 8). The high ratio ASD subgroup mainly revealed negative associations between ALR and spontaneously produced IL-1ß and IL-6. In contrast, the normal and low ratio ASD subgroups revealed positive associations between ALR and IL-1ß or IL-6 levels. MRC in the normal ratio ASD subgroup also showed similar results. The low ratio ASD subgroup revealed negative associations between ALR or MRC and levels of TNF-α under multiple culture conditions. It should be noted that associations between ALR or MRC and monocyte cytokine profiles revealed higher *p*-values (*p* < 0.005 or lower) in the normal ratio ASD subgroup.

**Table 8:** Associations between parameters of mitochondrial respiration (PLR, ALR, and MRC) and cytokine levels produced by PBMo.

**Cytokine showed correlation with PLR, ALR, and MRC*^a^***	**IL-1ß/IL-10 ratio^d^ based ASD subgroups**			**Non-ASD control cells (*N* = 38)**
	**High ratio (*N* = 56)**	**Normal ratio (*N* = 59)**	**Low ratio (*N* = 22)**	
**PLR^c^**				
IL-1ß (medium)^a^	−0.2875 (*p <* 0.05)^b^	0.1494	−0.0744	0.1741
IL-1ß (LPS)	−0.37 (*p <* 0.01)	−0.0795	−0.2572	0.1242
CCL2 (LPS)	0.324 (*p <* 0.02)	−0.2164	0.2668	0.1087
**ALR**				
IL-1ß (medium)	−0.422 (*p <* 0.001)	0.3825 (*p <* 0.005)	−0.0311	0.0988
IL-1ß (LPS)	−0.2535	0.4154 (*p <* 0.005)	0.1006	0.0551
IL-1ß (zymosan)	−0.155	0.1213	0.5139 (*p <* 0.02)	0.1864
IL-10 (LPS)	0.1696	0.3293 (*p <* 0.02)	0.0435	0.188
IL-6 (medium)	−0.3306 (*p <* 0.02)	0.4148 (*p <* 0.005)	0.0548	0.0755
IL-6 (LPS)	0.0111	0.3735 (*p <* 0.005)	0.1295	0.1902
IL-6 (zymosan)	0.0308	0.1863	0.5139 (*p <* 0.02)	0.207
TNF-α (medium)	−0.1248	0.0394	−0.6064 (*p <* 0.005)	−0.0422
TNF-α (LPS	−0.0696	0.0228	−0.5601 (*p <* 0.01)	−0.1003
TNF-α (zymosan)	−0.1351	−0.2718 (*p <* 0.05)	−0.2154	−0.3078
TNF-α (CL097)	0.1403	0.1842	−0.4354 (*p <* 0.05)	0.2563
TGF-ß (CL097)	0.0274	−0.077	0.5093 (*p <* 0.02)	−0.2786
**MRC^e^**				
IL-1ß (medium)	−0.2258	0.2971 (*p <* 0.05)	0.0184	−0.0078
IL-1ß (LPS)	−0.0302	0.524 (*p <* 0.0001)	0.1423	0.0119
IL-1ß (zymosan)	−0.1154	0.1638	0.5479 (*p <* 0.01)	0.1842
IL-10 (LPS)	0.2896 (*p <* 0.05)	0.4935 (*p <* 0.0001)	0.2101	0.0219
IL-6 (medium)	−0.0622	0.4286 (*p <* 0.001)	0.1209	−0.1224
IL-6 (LPS)	0.1178	0.4402 (*p <* 0.005)	0.3073	0.1286
TNF-α (medium)	−0.1054	0.0532	−0.5576 (*p <* 0.01)	−0.1702
TNF-α (LPS)	0.0072	0.1358	−0.4588 (*p <* 0.05)	−0.1959
TNF-α (zymosan)	−0.1528	−0.3308 (*p <* 0.02)	−0.1706	−0.3861 (*p <* 0.05)
TNF-α (CL097)	−0.1009	0.1082	−0.5800 (*p <* 0.005)	−0.3425
CCL2 (medium)	−0.1445	0.1689	0.0164	−0.4162 (*p <* 0.01)

a *Values of Correlation coefficients revealed significant results in at least one of ASD subgroups or non-ASD controls are shown. Stimulants used for cultures of PBMo are shown in the parentheses*.

b *Correlation coefficient by Spearman test; Statistically significant values are shown with p-values*.

c Abbreviations used: please see Table 3

d *Definition of high, normal, and low ratio groups are detailed in the method section*.

e *Correlations between RC and monocyte cytokine levels are almost identical as observed in MRC and monocyte cytokine levels. Thus, not shown in the table*.

Next, we analyzed associations between monocyte cytokine profiles and ALR/PLR, MR/PLR, and RC/PLR ratios, as markers of ETC efficiency, among the IL-1ß/IL-10 ratio based ASD subgroups. The associations between monocyte cytokine profiles and ETC efficiency markers differed across the IL-1ß/IL-10 ratio based ASD subgroups (Table 9). The high ratio ASD subgroup revealed positive associations between ETC efficiency parameters and IL-10 levels under LPS stimulated cultures. In the ASD normal ratio subgroup, ETC efficiency parameters were positively associated with IL-1ß levels and IL-1ß/IL-10 ratios under LPS stimulated cultures. In the low ratio ASD subgroup, positive associations were observed between ETC efficiency parameters and IL-1ß levels under zymosan-stimulated cultures. The most striking positive associations were observed between IL-1ß/IL-10 ratios (under LPS stimulated cultures) and MRC/PLR as well as RC/PLR ratios (*p* < 0.0005) in the normal ratio ASD subgroup. While the low ratio ASD subgroup revealed a negative association between MRC/PLR or RC/PLR and IL-1ß/IL-10 ratios (under CL097 stimulated cultures).

**Table 9:** Assessment of correlations between PBMo cytokine production and markers of ETC efficiency (ALR/PLR, MRC/PLR, and RC/PLR).

**Cytokine showed correlation with ALR/PLR, MRC/PLR, and RC/PLR^a^**	**IL-1ß/IL-10 ratio^d^ based ASD subgroups**			**Non-ASD controls (*N* = 38)**
	**High ratio (*N* = 56)**	**Normal ratio (*N* = 59)**	**Low ratio (*N* = 22)**	
**ALR/PLR^c^**				
Ratio (LPS)	**-**0.1907^b^	−0.359 (*p <* 0.01)	0.4033	**–**0.0631
Ratio (zymosan)	−0.3285 (*p <* 0.02)	−0.0309	0.0932	0.2167
IL-1ß (LPS)	−0.0384	0.3122 (*p <* 0.02)	0.3303	−0.1191
IL-1ß (zymosan)	0.0289	0.0965	0.4783 (*p <* 0.05)	0.0379
IL-10 (LPS)	0.3468 (*p <* 0.01)	0.0506	0.2298	0.1267
**MRC/PLR**				
Ratio (LPS)	−0.1415	0.473 (*p <* 0.0005)	0.2569	−0.1251
Ratio (CL097)	−0.0679	−0.0954	−0.5089 (*p <* 0.02)	−0.0501
IL-1ß (LPS)	0.0019	0.392 (*p <* 0.005)	0.2976	−0.1587
IL-1ß (zymosan)	0.0267	0.1176	0.5088 (*p <* 0.02)	0.0333
IL-10 (LPS)	0.356 (*p <* 0.01)	−0.024	0.2863	0.0123
**RC/PLR**				
Ratio (LPS)	**–**0.1201	0.5029 (*p <* 0.0005)	0.1836	−0.1067
Ratio (CL097)	−0.0677	0.1154	−0.5345 (*p <* 0.01)	−0.0992
IL-1ß (LPS)	0.025	0.4204 (*p <* 0.005)	0.2677	−0.1417
IL-1ß (zymosan)	0.0355	0.1225	0.4607 (*p <* 0.05)	−0.0355
IL-10 (LPS)	0.3504 (*p <* 0.01)	0.0377	0.3321	−0.0278

a *The relationship between levels of cytokine produced by PBMo and ALR/PLR, MRC/PLR, or RC/PLR. Results are shown in cytokines that showed positive or negative associations with ETC efficiency parameters in at least one of ASD subgroups or non-ASD controls. Stimulants used for PBMo cultures are shown in the parentheses. The details of PBMo culture conditions are shown in the method section*.

b *Correlation coefficient by Spearman Test. When the results are significant, the values are shown with p-values*.

c *Abbreviations used: please see Table 3*.

d *Definition of high, normal, and low ratio groups are detailed in the method section*.

## Discussion

The results of our results indicate associations exist between mitochondrial respiration by PBMCs and monocyte cytokine profiles in ASD subjects. Our findings may be the result of the presence of ASD subjects in whom adaptive changes triggered by environmental stimuli are dysregulated in both innate immunity and in mitochondrial function. In such subjects, maladapted changes may cause detrimental effects on the nervous system, leading to a puzzling array of clinical features in ASD subjects.

Mounting evidence indicates that there is abnormal or altered mitochondrial function in individuals with ASD (5, 28). There is also mounting evidence of mitochondrial dysfunction and chronic oxidative stresses in ASD (11–14). Interestingly, individuals with ASD seem to have unique types of mitochondrial dysfunction. For example, while it is estimated that 5% of individuals with ASD appear to have primary mitochondrial disease, the majority (~75%) do not have known genetic mutations to explain their mitochondrial disease (5, 15). What is more interesting is that 30% of more individuals with ASD have biomarkers of mitochondrial dysfunction, even though they may not have primary mitochondrial disease (16). In addition, up to 80% of immune cells (lymphocytes and granulocytes) may show abnormalities in the respiratory chain when examined (29, 30). Furthermore, converging evidence from several human tissues (lymphoblastic cell lines, buccal endothelium muscle, fibroblasts, and postmortem brain) demonstrate that the mitochondria may have atypical over-activity of the respiratory chain which may result in a vulnerability to oxidative insults (16). References of mitochondrial dysfunction in ASD subjects are summarized in Supplemental Table 2. One important unanswered question is the reason for these alterations in mitochondrial function, especially given the fact that no clear genetic cause seems to explain these changes.

Apart from changes in mitochondrial functions, immune abnormalities are also frequently reported in ASD children (31–35). Although immune abnormalities reported in ASD subjects affect almost every arm of the immune system, multiple researchers have reported innate immune abnormalities independently. This may not be surprising, since one of the most studied animal models of autism is the maternal immune activation (MIA) in rodents; in this model, sterile inflammation triggered by stimuli of innate immunity during pregnancy results in aberrant behaviors and impaired cognitive activity in offspring (36–39).

The findings in MIA models indicate that during critical period of pregnancy (2nd trimester), maternally derived inflammatory mediators affect developing fetal brain, resulting in ASD like behaviors and impaired cognitive activity in offspring (38, 40). Interestingly, several studies have linked the MIA model with mitochondrial dysfunction and abnormalities in redox metabolism (41–43). Therefore, it may be possible that changes in innate immune responses are inter-related with mitochondrial dysfunction and together, they impose detrimental effects on neurodevelopment in ASD children. Our previous study has also shown that changes in cytokine profiles of monocytes, major innate immune cells in the periphery, are associated with changes in behavioral symptoms in some ASD subjects (8).

Activation of innate immunity is known to shape subsequent changes in adaptive immunity via cell-cell interactions, as well as soluble mediators. Antigens (Ags) are typically taken up by innate immune cells, processed, and presented to T cells. Ag-triggered T cell activation is tightly regulated to avoid excessive immune responses. Recent research highlights the importance of metabolic control of the immune system in fine tuning of the immune responses. That is, upon immune activation, effector T cells are required to proliferate rapidly, preferentially producing ATP through glycolysis, while regulatory T (Treg) cells with anti-inflammatory natures require the generation of mitochondrial ATP (9). Therefore, adaptive changes in mitochondrial function in effector immune cells in ASD subjects could result in immune imbalances, postulated to be present in our hypotheses. In this study, we also postulated that metabolic changes reflected in changes in mitochondrial function of immune cells are associated with changes in cytokine profiles by innate immune cells, such as monocytes.

In previous studies, we found that changes in IL-1ß/IL-10 ratios produced by PBMo from ASD subjects are closely associated with changes in miRNA expression in ASD PBMo (4). Namely, significant up-regulation of multiple miRNAs were observed in the high IL-1ß/IL-10 ratio ASD subgroup, while normal and low ratio ASD subgroups revealed down-regulation of multiple miRNAs, as compared to non-ASD control PBMo (4). Such changes were less evident when miRNA expression by ASD PBMo as a whole was compared to non-ASD controls. We also analyzed targeted genes by these miRNAs that revealed differences in expression in the IL-1ß/IL-10 based ASD subgroup. The results revealed that such changes in miRNA expression would modulate immune cell functions and mitochondrial fitness through several pathways, including mTOR-PI-3K pathways in the high or low ratio ASD subgroups (4). Interestingly, gene mutations in these pathways have also revealed an association between immune and mitochondrial dysfunction. Specifically, in a PTEN hamartoma tumor syndrome, deficiency of PTEN causes alteration in the mTOR-PI3K pathway, resulting in dysregulated T cell activation and impaired mitochondrial fitness (44). Moreover, patients with PTEN mutation exhibit a broad spectrum of neuropsychiatric syndromes, including ASD (45). Indeed, a new ASD mouse model was created by inducing germline mislocalization of PTEN (46). Taken together, it may be postulated that abnormalities found in ASD PBMo are closely associated with mitochondrial dysfunction reported in some ASD subjects.

As briefly discussed in the introduction section, miRNAs are known to serve as mediators of innate immune cells, affecting functions of other immune and even non-immune cells not located in close proximity (10). This is because miRNAs secreted by secretary cells like monocytes are stable as a form of exosomal miRNA and circulate in the body fluid. They affect functions of other lineage cells when taken up. In our other study, we have already identified serum levels of miRNAs that are significantly altered across the IL-1ß/IL-10 based ASD subgroups (preliminary results and manuscript submitted for publication). Therefore, we postulated, if miRNAs serve as mediators of innate immune responses to other lineage cells, we will be able to detect associations between monocyte cytokine production profile and mitochondrial functions of PBMCs, a mixture of immune cells including lymphocytes, monocytes, dendritic cells, and natural killer cells.

First, we determined whether there is any evidence of adaptive changes in mitochondrial function in ASD PBMCs. Consistent with the previous report in transformed B lymphoblastoid cell lines derived from ASD subjects (14), we observed higher levels of MRC and RC in ASD PBMCs (Figure 1). We also observed altered association between PLR and ALR in ASD PBMCs. The expected positive association between PLR and ALR was lost in ASD PBMCs, due to the presence of ASD cells with high ALR despite low PLR, and those with low ALR despite high PLR. High ALR with low PLR is considered to reflect efficient ATP production, most likely reflecting adapted changes in mitochondrial function in response to chronic oxidative stresses, but this may lead to increase in production of ROS. While low ALR with increase in PLR indicates mitochondrial failure, being unable to compensate on-going oxidative stresses. These results indicate that a fair number of ASD PBMCs reveals changes in mitochondrial function that may reflect dysregulation of normal control mechanism, perhaps as consequences of adaptive changes and/or failure of mitochondrial function. Since ASD subjects recruited to this study were those without any known gene mutations, these changes are more likely to reflect physiological and/or pathological alterations in the regulatory pathways.

We then determined whether observed changes in mitochondrial functions in ASD PBMCs parallel changes in cytokine profiles produced by PBMo. In select ASD subjects, we simultaneously assessed mitochondrial function in PBMCs and monocyte cytokine production by PBMo at 2–3 time points. Similar to our previous time-course study (4, 8), we found variable levels of PLR, ALR, MRC, and RC in some ASD subjects, while these levels remained stable in others (Figure 3). Our results may indicate a possibility of a positive association between mitochondrial respiration and IL-1ß/IL-10 ratios in some ASD subjects, although further studies are necessary with the use of samples taken at multiple time points in a larger number of study subjects.

We then analyzed associations between ASD samples and monocyte cytokine profiles between ASD samples as a whole and non-ASD controls. Our results revealed some associations between these two groups of parameters, but these associations were not strong, partly reflecting variable values of mitochondrial parameters as revealed in Figures 1, 2. Since we did not find close associations between mitochondrial parameters and monocyte cytokine profiles in non-ASD controls either, these two variables may not be associated, not supporting our initial hypothesis. On the other hand, as detailed in the 2nd paragraph of the Discussion section, apparent unique mitochondrial dysfunction observed in ASD subjects may be associated with chronic oxidative stress mediated by immune mediated inflammation in some ASD subjects. To further address such a possibility, we turned assessing changes in mitochondrial parameters in the IL-1ß/IL-10 based ASD subgroups, since our previous results indicated that IL-1ß/IL-10 ratios are closely associated with behavioral changes and miRNA expression in ASD PBMo in our previous studies (4, 8).

Our results have shown that ASD PBMCs revealed higher MRC and RC, only when ASD PBMo revealed high or normal IL-1ß/IL-10 ratios (Table 5). We also determined whether ALR/PLR, MRC/PLR, and RC/PLR ratios, as markers of ETC efficiency, are altered in the IL-1ß/IL-10 ratio based ASD subgroups. Our results revealed differences in RC/PLR between specific ASD subgroups and non-ASD controls: these ratios were higher only in the high and normal IL-1ß/IL-10 ratio ASD subgroups than controls (Table 6). High RC/PLR may indicate efficient mitochondrial function or low generation of mitochondrial oxidative stress. However, given lack of expected positive associations between ALR and PLR in ASD subjects, increase in RC/PLR is more likely secondary to an adaptive increase in mitochondrial activity. Taken together, changes in RC/PLR ratios in the IL-1ß/IL-10 ratio based ASD subgroups indicated a possibility, that changes in monocytes cytokine profiles may affect mitochondrial function of PBMCs in ASD. Alternatively, this finding may indicate changes in regulatory mechanisms of immune metabolism affecting both PBMCs and PBMo in ASD. When we assessed associations between mitochondrial parameters and IL-1ß/IL-10 ratios under different culture conditions, we also observed differences of associations between these two groups of parameters across the IL-1ß/IL-10 based ASD subgroups (Table 7).

Cytokines produced by monocytes exert multiple functions and their actions are closely inter-related and they are often categorized as “proinflammatory” vs. “counter-regulatory” cytokines. Although we have used IL-1ß/IL-10 ratios as a surrogate marker for balanced immune responses, mitochondrial functions can also be affected by other monocyte cytokines. We, therefore, further determined if mitochondrial respiration in PBMCs were associated with representative proinflammatory (IL-1ß, IL-6, and TNF-α) and counter-regulatory (IL-10, TGF-ß, and TNFRII) cytokine levels produced by PBMo.

When associations between parameters of mitochondrial respiration (PLR, ALR, MRC, and RC) and cytokine levels produced by PBMo were examined, we also observed marked differences across the IL-1ß/IL-10 based ASD subgroups (Table 8). Namely, only the high ratio ASD subgroup revealed positive associations between PLR and IL-1ß. The above described results may indicate a compensatory increase in protein leak to reduce ROS with increase in production of IL-1ß, an inflammatory cytokine, in the high ratio ASD subgroup.

On the other hand, the normal ratio ASD subgroup revealed positive associations between ALR and MRC with IL-1ß, IL-6, and IL-10 levels. This finding may indicate that adapted mitochondrial responses are on-going in response to changes in innate immune responses. In the low ratio ASD subgroup, levels of TNF-α under multiple culture conditions revealed negative associations with ALR and MRC. TNF-α is a major inducer of apoptosis and autophagy, affecting mitochondrial functions (47). Inappropriate mitochondrial responses to TNF-α is associated with dysregulated apoptosis or clearance of cell organelles, leading to pathological conditions such as tumorigenesis (47). PBMCs in the low ratio ASD subgroup may be in the state of mitochondrial dysfunction, not adapting to changes in monocyte cytokine production. Such associations are not revealed in control non-ASD subjects. Since we found a high frequency of history of NFA in ASD subjects whose monocyte revealed low IL-1ß/IL-10 ratios than the normal ratio ASD subgroup (Table 4), a major source of chronic oxidative stress may be gut inflammation in these ASD subjects.

We then assessed associations between ETC efficiency parameters (ALR/PLR, MRC/PLR, and RC/PLR ratios) and cytokine levels produced by PBMo. Our results revealed that associations between parameters of ETC efficiency and monocyte cytokine levels produced by PBMo again markedly differed across the IL-1ß/IL-10 ratio based ASD subgroups (Table 9). Namely, the IL-1ß/IL-10 high ratio group revealed a positive association between ETC efficiency parameters and IL-10 levels produced by PBMo under LPS stimulated cultures. Given the fact that IL-10 is a counter-regulatory cytokine, it may be speculated that in the high ratio ASD subgroup, suppressive mechanisms may be in place. In contrast, the normal and low ratio ASD subgroups revealed positive associations between ETC efficiency markers and IL-1ß levels. IL-1ß is an inflammatory cytokine and implicated in the stress responses to the brain. Thus, in these two ASD subgroups, up-regulatory drive for ETC efficiency may be in place.

Taken together, the above described results indicate that associations between monocyte cytokine profiles and mitochondrial parameters in PBMCs differed significantly across the IL-1ß/IL-10 based ASD subgroups, and these changes also differed from non-ASD controls. Our findings support our initial hypothesis that innate immune abnormalities and mitochondrial abnormalities, two abnormalities frequently reported in ASD subjects, are closely inter-related and may exert detrimental effects on the brain. However, our results did not clarify whether innate immune responses cause secondary mitochondrial dysfunction or dysregulations of common pathways key to functions of innate immunity and mitochondrial respiration. It remains to be seen how these changes are associated with ASD clinical features and health outcomes in future studies.

Although many questions need to be answered in further studies, our results indicate that concurrent use of immune-modulating agents and mitochondrial rescue medications may be required in ASD subjects who exhibit innate immune abnormalities and mitochondrial dysfunction, possibly due to alterations in the signaling pathways affecting the both systems. In each such ASD individual, fine adjustment in doses and administration schedule will be required.

## Author Contributions

HJ was responsible for the study design, recruitment of the study subjects, collection of clinical information, and blood samples, analysis of the overall data, and preparation of most of this manuscript. LG conducted cytokine production assays with the use of purified monocytes and also prepared samples for mitochondrial function for shipping to RF's laboratory. She also adopted biofreezing methodology of PBMCs. SR and SB conducted assays for mitochondrial respiration with the use of PBMCs and helped prepare a manuscript. RF supervised SR and SB and discussed with HJ extensively, regarding data analysis, and helped extensively for manuscript preparation.

### Conflict of Interest Statement

The authors declare that the research was conducted in the absence of any commercial or financial relationships that could be construed as a potential conflict of interest.

## Abbreviations

Ab: antibody
ABC: aberrant behavior checklist
AC: allergic conjunctivitis
ADI-R, autism diagnostic inventory: revisited
ADOS: autism diagnostic observational scale
Ag: antigen
Akt: protein kinase B activated through PI3K-Akt pathway
ALR: ATP-linked respiration
ANOVA: analysis of variance
AR: allergic rhinitis
ASD: autism spectrum disorder
CSHQ: Children's sleep habit questionnaire
ELISA: enzyme linked immune-sorbent assay
ETC: electron transport chain
FA: food allergy
IL: interleukin
MIA: maternal immune activation
miRNA: microRNA
MRC: maximum respiration capacity
NFA: non-IgE mediated food allergy
OCR: oxygen consumption rate
PBMCs: peripheral blood mononuclear cells
PBMo: peripheral blood monocytes
PI3K: phosphoinositide 3-kinase
PTEN: phosphatase and tension homolog
PLR: proton-linked respiration
PST: prick skin testing
RC: reserve capacity
SAD: specific antibody deficiency
SD: standard deviation
TGF: transforming growth factor
TLR: toll like receptor
TNF: tumor necrosis factor
Treg cells: regulatory T cells
VABS: Vineland adaptive behavioral scale.
