# Supplementary material for: Variations in Mitochondrial Respiration Differ in IL-1ß/IL-10 Ratio Based Subgroups in Autism Spectrum Disorders
Source: Front Psychiatry. 2019 Feb 20;10:71. doi: 10.3389/fpsyt.2019.00071 (PMC6391925; doi:10.3389/fpsyt.2019.00071)
Supplement: Supplementary file 1 [file Table_1.docx]

**Suppl. Table 1 Monocyte cytokine profiles by ASD and non-ASD control PBMo**

| Cytokines | IL-1ß/IL-10 ratio based ASD subgroups | | | non-ASD  controls  (N=38) | Krushkal Wallis test |
| --- | --- | --- | --- | --- | --- |
|  | High  (N=56) | Normal  (N=59) | Low  (N=22) |  |  |
| IL-6 | | | | | |
| medium | 2363 ± 1673^1^ | 2374 ± 1567 | 1394 ± 1036 | 2770 ± 2022 | 0.0297^2^ |
| LPS | 27000 ± 28646 | 24043 ± 13136 | 22178 ± 15451 | 22419 ± 11614 | 0.6201 |
| Zymosan | 6050 ± 1892 | 6127 ± 1754 | 4887 ± 2406 | 6624 ± 1585 | 0.0112 |
| CL097 | 6123 ± 6223 | 5344 ± 2300 | 6000 ± 2269 | 6147 ± 2391 | 0.2212 |
| TNF-α | | | | | |
| medium | 71.4 ± 171.5 | 60.2 ± 184.8 | 38.9 ± 88.9 | 69.0 ± 100.0 | 0.2154 |
| LPS | 244.4 ± 336.0 | 226.8 ± 439.4 | 262.8 ± 320.5 | 290.4 ± 370.1 | 0.5824 |
| Zymosan | 860.6 ± 749.1 | 664.8 ± 753.5 | 741.3 ± 915.2 | 1186.9 ± 969.4 | 0.0124 |
| CL097 | 834.6 ± 749.1 | 842.2 ± 1123.9 | 1469.2 ± 1189.2 | 1379.5 ± 1298.5 | 0.0313 |
| CCL2 | | | | | |
| medium | 10182 ± 7531 | 19372 ± 6823 | 15851 ± 8049 | 21069 ± 7443 | 0.069 |
| LPS | 14804 ± 6518 | 14557 ± 6698 | 13725 ± 8600 | 15457 ± 9107 | 0.8163 |
| Zymosan | 8516 ± 5279 | 10874 ± 5679 | 10758 ± 6824 | 9780 ± 8060 | 0.072 |
| CL097 | 17919 ± 11119 | 19935 ± 9890 | 19888 ± 9407 | 19461 ± 9868 | 0.7459 |

**^1^** Results are expressed as mean ± SD ng/mL

**^2^** These p values are nominally significant (p<0.05), but not significant by Bonferroni correction (α=0.0025).

**Suppl. Table 1**. Monocyte cytokine profiles in the IL-1ß/IL-10 based ASD subgroups and non-ASD controls. Consistent with our previous results, we also found differences in IL-6 and TNF-α production by PBMo in these subgroups. CCL2 also revealed differences but these results were not statically significant by Krushkal Wallis test.
