# Supplementary material for: Variations in Mitochondrial Respiration Differ in IL-1ß/IL-10 Ratio Based Subgroups in Autism Spectrum Disorders
Source: Front Psychiatry. 2019 Feb 20;10:71. doi: 10.3389/fpsyt.2019.00071 (PMC6391925; doi:10.3389/fpsyt.2019.00071)
Supplement: Supplementary file 2 [file Table_2.DOCX]

**Supp. Table 2. Summary of references describing mitochondrial abnormalities in ASD**

| Mitochondrial abnormalities in ASD | References |
| --- | --- |
|  |  |
| Oxidative stress and mitochondrial dysfunction in ASD  (abnormalities in redox metabolism) | Rossignol and Frye 2012a (Review)  Frye and James 2014 (Review) |
| Tissue or cell lineage specific mitochondrial dysfunction in ASD  Lymphoblastoid cell line    Peripheral blood lymphocyte  Granulocytes  Gastrointestinal mucosa  Other tissues | Frye et al 2017, Rose et al 2014,  Rose et al. 2017c  Giulivi et al. 2010 (not seahorse assay)  Napoli et al. 2014  Rose et al. 2017b  Rose et al. 2018 (Review) |
| Primary mitochondrial disease in ASD (rare) | Rossignol and Frye 2012a (Review)  Marin and Saneto 2016 |
| Frequency of mitochondrial dysfunction in ASD | Rose et al. 2018 (Review) |
| Oxidative stress specific for ASD diagnosis | Howsmon et al. 2018 |
| MIA model (Animal model of autism)  Abnormalities in redox metabolism | Guilivi, et al. 2013,  Naviaux et al. 2013 |

**Suppl. Table 2.** Summary of previous publications describing mitochondrial abnormalities in ASD subjects and in a rodent model of autism (MIA).
