# Supplementary material for: Variations in Mitochondrial Respiration Differ in IL-1ß/IL-10 Ratio Based Subgroups in Autism Spectrum Disorders
Source: Front Psychiatry. 2019 Feb 20;10:71. doi: 10.3389/fpsyt.2019.00071 (PMC6391925; doi:10.3389/fpsyt.2019.00071)
Supplement: Supplementary file 3 [file Data_Sheet_1.PDF]

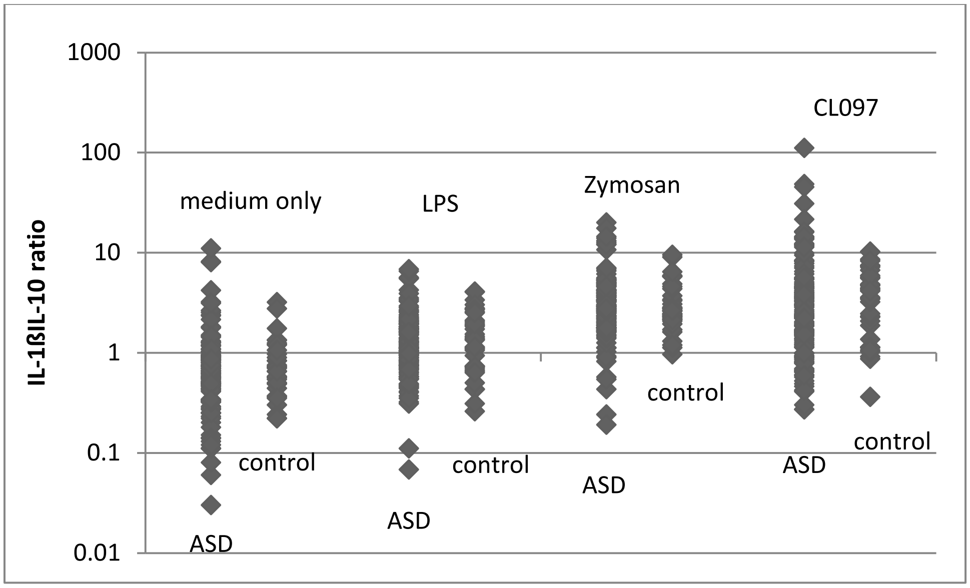

Supplemental Fig. 1 IL-1ß/IL-10 ratios produced by ASD or non-ASD control monocytes under culture conditions with medium only, or stimulated with LPS, zymosan, or CL097**.** F-ratio for medium only, and for cultures stimulated with LPS, zymosan, and CL097 are 0.10139, 0.3732, 1.7402, and 0.4714, respectively (not significant) by Welch's test.
