# Supplementary material for: Variations in Mitochondrial Respiration Differ in IL-1ß/IL-10 Ratio Based Subgroups in Autism Spectrum Disorders
Source: Front Psychiatry. 2019 Feb 20;10:71. doi: 10.3389/fpsyt.2019.00071 (PMC6391925; doi:10.3389/fpsyt.2019.00071)
Supplement: Supplementary file 4 [file Data_Sheet_2.PDF]

Numerical data
Comparison between 2 variables → Two tailed Mann Whitney test (Tables 2, 5, 6)
Comparison between >2 variables → Krushkal Wallis test (Tables 5, 6)

Associations between 2 variables → Spearman test (Tables 3, 7, 8, 9)

Co-variance of repeated measures between 2 variables →regression analysis (mixed models – repeated measures (Fig. 3)

Frequencies
Comparison >2 groups → Qui Square test (Table 4)
Comparison between 2 groups →Fisher's exact test (Table 4)

Supplemental Fig. 2 Summary of statistical measures used in this study
